# Transmission and Demographic Dynamics of Coxsackievirus B1

**DOI:** 10.1371/journal.pone.0129272

**Published:** 2015-06-08

**Authors:** Pei-Yu Chu, Yu-Chang Tyan, Yao-Shen Chen, Hsiu-Lin Chen, Po-Liang Lu, Yu-Hsien Chen, Bao-Chen Chen, Tsi-Shu Huang, Chu-Feng Wang, Hui-Ju Su, Yong-Ying Shi, Bintou Sanno-Duanda, Kuei-Hsiang Lin, Kazushi Motomura

**Affiliations:** 1 Department of Medical Laboratory Science and Biotechnology, College of Health Sciences, Kaohsiung Medical University, Kaohsiung, Taiwan, ROC; 2 Department of Laboratory Medicine, Kaohsiung Medical University Hospital, Kaohsiung, Taiwan, ROC; 3 Department of Medical Imaging and Radiological Sciences, Kaohsiung Medical University, Kaohsiung 807, Taiwan; 4 Translational Research Center, Kaohsiung Medical University Hospital, Kaohsiung 807, Taiwan; 5 National Sun Yat-Sen University-Kaohsiung Medical University Joint Research Center, Kaohsiung 804, Taiwan; 6 Institute of Medical Science and Technology, National Sun Yat-Sen University, Kaohsiung 804, Taiwan; 7 Division of Infectious Diseases, Kaohsiung Veterans General Hospital, Kaohsiung, Taiwan, ROC; 8 Division of Microbiology, Department of Pathology and Laboratory Medicine, Kaohsiung Veterans General Hospital, Kaohsiung, Taiwan, ROC; 9 Department of Internal Medicine, National Yang-Ming Medical University, Taipei, Taiwan, ROC; 10 Department of Pediatrics, Kaohsiung Medical University Hospital, Kaohsiung, Taiwan; 11 Department of Respiratory Therapy, College of Medicine, Kaohsiung Medical University, Kaohsiung, Taiwan; 12 Department of Laboratory Medicine, School of Medicine, College of Medicine, Kaohsiung Medical University, Taiwan; 13 Edward Francis Small Teaching Hospital, Banjul, Gambia; 14 Pathogen Genomics Center, National Institute of Infectious Diseases, Tokyo, Japan; 15 Thailand-Japan Research Collaboration Center on Emerging and Re-emerging Infections, Research Institute of Microbial Diseases, Osaka University, Nonthaburi, Thailand; British Columbia Centre for Excellence in HIV/AIDS, CANADA

## Abstract

The infectious activity of coxsackievirus B1 (CV-B1) in Taiwan was high from 2008 to 2010, following an alarming increase in severe neonate disease in the United States (US). To examine the relationship between CV-B1 strains isolated in Taiwan and those from other parts of the world, we performed a phylodynamic study using VP1 and partial 3D^pol^ (414 nt) sequences from 22 strains of CV-B1 isolated in Taiwan (1989–2010) and compared them to sequences from strains isolated worldwide. Phylogenetic trees were constructed by neighbor-joining, maximum likelihood, and Bayesian Monte Carlo Markov Chain methods. Four genotypes (GI–IV) in the VP1 region of CV-B1 and three genotypes (GA–C) in the 3D^pol^ region of enterovirus B were identified and had high support values. The phylogenetic analysis indicates that the GI and GIII strains in VP1 were geographically distributed in Taiwan (1993–1994) and in India (2007–2009). On the other hand, the GII and GIV strains appear to have a wider spatiotemporal distribution and ladder-like topology A stair-like phylogeny was observed in the VP1 region indicating that the phylogeny of the virus may be affected by different selection pressures in the specified regions. Further, most of the GI and GII strains in the VP1 tree were clustered together in GA in the 3D tree, while the GIV strains diverged into GB and GC. Taken together, these data provide important insights into the population dynamics of CV-B1 and indicate that incongruencies in specific gene regions may contribute to spatiotemporal patterns of epidemicity for this virus.

## Introduction

Enterovirus (EV) outbreaks caused by coxsackievirus B1 (CV-B1) are rare, and most CV-B1 infections are subclinical. However, CV-B1 reportedly has a strong association with systemic neonatal infections, including meningoencephalitis, myocarditis, sepsis, and hepatitis, all of which can rapidly deteriorate to critical status [1, 2]. Infection with CV-B1 is also a suspected risk factor for insulin-dependent diabetes mellitus and polyomyositis [3–6]. In regards to outbreak circulation pattern, CV-B1 infection is uniquely characterized by prominent increases in circulatory activity, which usually last 2–3 years, but occur at irregular intervals [7]. Increased CV-B1 activity has been reported in the United States (US; 2007–2008) and in South Korea (2008–2009), and has been associated with severe infections in young infants in both countries [8, 9]. Although large CV-B1 outbreaks are rare, this serotype was among the five most active enteroviruses in Taiwan during 1993–1994, in 1999, and during 2008–2010 [10–12]. Moreover, EV outbreaks are known to occur annually in tropic area, and different serotypes may co-circulate with widely fluctuating prevalence. For example, a sudden large outbreak in one genotype may be followed by period of dormant infectivity due to herd immunity. In contrast, irregular outbreaks or short dormant periods often indicate the emergence of a new variant [13, 14]. Therefore, it is important to use molecular epidemiological surveillance to help identify prevalent emerging strains and forecast trends in viral circulation.

Human enterovirus B was renamed enterovirus B (EV-B) in 2013 [15], and CV-B1 is a serotype of the EV-B species, in the family *Picornaviridae*. The VP1 gene in the EV family encodes the major serotype-specific neutralization epitopes present on the capsid, and its sequence strongly correlates with the major serotype classifications [16, 17]. Furthermore, recombination, which is reportedly a common phenomenon in the EV-B family, can be recognized by identifying genotypic incongruencies in the VP1 and 3D^pol^ gene regions [18–20]. The 3D^pol^ gene encodes RNA dependent RNA polymerase (RdRp), which is essential for RNA synthesis in most RNA viruses. Rather than sequence variation of 3D region differ by serotype, the emergence of the 3D^pol^-based clusters reportedly correlates with the time of virus isolation [21, 22] implying that researchers may be able to use VP1 and 3D^pol^ gene sequences to track the trajectories of evolution in EV. This is particularly useful as sequence variations, including mutations and recombination events, are implicitly stochastic, occur at different frequencies, and involve different virus types, allowing each change to be followed through viral transmission history.

Phylodynamic analyses have been previously used to elucidate epidemic episodes in viral evolution and transmission [23]. For example, if a viral strain causing an outbreak in the US is clustered together with a strain that was active in Taiwan during 2008–2010 with high sequence similarity and high node support values, then it is likely that the two outbreak events and strains are associated. Therefore, to identify relationships among CV-B1 strains in Taiwan and elsewhere in the world, we reconstructed the spatiotemporal transmission and demographic history of this specific virus by performing a phylodynamic sequence analysis of two gene regions, VP1 and 3D^pol^.

## Materials and Methods

### Specimen collection and ethics statement

For each year of positive CV-B1 isolations (1989–2010), this study randomly selected 22 samples isolated at one of the two medical centers in southern Taiwan (Kaohsiung Veterans General Hospital or Kaohsiung Medical University Hospital). This study was performed according to the principles expressed in the Declaration of Helsinki and was approved by the ethics committees of both hospitals. All samples were de-identified and analyzed anonymously.

### Viral RNA extraction, RT-PCR, and sequencing

Confluent Rhabdomyosarcoma (RD) cells were used to amplify virus strains. RNA purification and sequencing were performed as previously described [13]. Table 1 shows the primer sets used to amplify PCR fragments. Amplifications of the VP1 and 3D^pol^ regions were performed separately, and two researchers independently confirmed all results. The sequences obtained from the 22 Taiwan isolates of the full length VP1 gene (834 nucleotide; nt) and part of the 3D^pol^ gene (411 nt, position 6682–6741 of accession no. M16560) were submitted to GenBank under the accession numbers AB639774−AB639793 and AB646477−AB646500.

**Table 1:** Primers used to amplify VP1 and 3Dpol regions of the Coxsackievirus B1.

Primer	Position ^a^	Sequence	Gene region	References
2400F	2372–2393	GCTTTGTGTCTGCMTGYAATGA	VP1	CDC-TW
5VP1F	2392–2410	GAYTTCTCWGTGCGGATGC	VP1	This study
292F	2546–2561	MIGCIGYIGARACNGG	VP1	[24]
222R	2882–2903	CICCIGGIGGIAYRWACAT	VP1	[24]
011R	3408–3389	GCICCIGAYTGITGICCRAA	2A	[24]
PY-CV-B1F	2794–2813	GARYTGACRTTTGTGGTAAC	VP1	This study
Chu 477F	2669–2688	GTGTWTAYTAYGCYACYTAC	VP1	This study
CVB2AR	3379–3395	TTRTAAYYYTCCCACAC	2A	This study
3DpolF	6681–6702	TTGAYTACWCWGGNTATGATGC	3D	[25]
3DpolR	7201–7191	WGSRTTCTTKGTCCATC	3D	[25]

^a^ Numbering system used in the Coxsackievirus B1 strain (Accession No. M16560).

### Multiple sequences alignment, model selection, and variation detection

Multiple alignments were created from BLAST results by using T-coffee program [26]. The resulting alignments were then manually corrected, and gap regions were removed. Strains with nonsense mutations were also excluded in the absence of independent laboratory verification. Sequences were stratified evenly by isolation year and location. In the VP1 region, all sequences with lengths close to the full length of CV-B1 VP1 were included, but sequences isolated in the same years and locations were randomly excluded. The resulting sequence dataset used in the VP1 analysis included 77 VP1 sequences. Of these, 22 were Taiwan strains isolated in this study. Prototype strains of CV-B3 were used as the outgroup. The dataset without the outgroup was used to reconstruct the demographic and spatiotemporal transmission. Since the nonstructural region in enterovirus is not monophyletic by serotype, intertypic recombination during the evolution of enterovirus is common [19, 27]. In the partial 3D region, a 133 sequences were sampled for phylogenetic analysis. This dataset included 22 sequences isolated in Taiwan and 111 sequences isolated worldwide (14 were CV-B1, and 111 were other serotypes). The worldwide sequences were chosen from 500 sequences obtained by a BLAST search of GenBank. All BLAST sequences belonged to EV-B. After manually correcting and excluding sequences with nonsense mutation, the CV-B1 sequences were sampled as the VP1 region. Other than CV-B1, no more than three strains in each genotype were chosen for recombination detection and phylogenetic analysis.

The most suitable nucleotide substitution model was identified the jModelTest v2.1.7 [28]. The model with the best fit was then used for recombination, phylogenetic and selection analyses. A four-category Tamura-Nei model [29] with the shape parameter of a gamma distribution (TN+G, G = 0.2070) was chosen as the best-fit model for the VP1 gene, while a general time reversible model [30] with gamma distribution and invariant sites (GTR+G+I) was chosen as the best-fit model for the 3D^pol^ sequences (G = 2.2855, I = 56.2832%). These models were then used in our phylogenetic analysis to detect recombination by neighbor-joining (NJ) and maximum likelihood (ML) methods. For Bayesian Markov Chain Monte Carlo (BMCMC) tree reconstruction, a model combining SRD06 substitution, the uncorrelated exponential model, and the Bayesian Skyline Plot (BSP) had the best support in Bayesian factor (BF) analysis in both the VP1 and 3D^pol^ datasets in analyses performed with Tracer v1.6. Pairwise comparison in nt and amino acid (aa) was detection by p-distance in MEGA6 [31]. Potential recombinant sequences were detected by using the Recombination detection program (RDP) v3.44 [32] and the Simplot v3.5.1 [33] software packages. The cutoff *p* value was set to greater than 0.05. Possible recombination events were detected with a series of algorithms in the RDP program, including RDP, GENECONV, BootScan, Maxchi, Chimaera, SiSscan, PhylPro, LARD, and 3Seq. The percentage of permutation and percentage similarity in a sliding window across the query sequence was compared to that in the reference sequences and plotted with the Simplot program. A range of windows and step sizes was used. The recombination relationships were further analyzed in a Kimura-2-parameter model using MEGA software to construct an NJ tree. Support values were tested in 1000 bootstrap (BS) iterations.

### Phylogenetic and phylodynamic analyses

Both phylogenetic and phylodynamic analyses were performed as previously described [13]. Briefly, the NJ and ML trees were constructed with MEGA6 software. The nodal reliability was assessed using the BS method with a significant support value greater than 70%. A BMCMC analysis was performed with the Bayesian Evolutionary Analysis by Sampling Tree (BEAST) v.1.8.1 program [34]. The nodal reliability of the MCMC trees were estimated by posterior probability (PP) with a significant support value greater than 0.9. A discrete phylogeographic analysis was used to infer the most important epidemiological links to CV-B1 [35]. The BEAST program was also used to estimate nucleotide substitution, population change history, and the time to the most recent common ancestor (TMRCA) [34]. The Tracer v.1.6 program was used to calculate effective sample size (ESS) for all estimated parameters. Convergence of the MCMC sample on the posterior distribution was defined by having an ESS value greater than 200. The summarized maximum clade credibility (MCC) tree was visualized using FigTree v.1.3.1 and converted to a keyhole markup language (KML) file with the SPREAD program [36]. A BF test was performed to obtain statistical data that adequately explained the phylogeographic process. The transmission pathways were then visualized in ArcGIS explorer (Environmental Systems Research Institute, Redlands, California, USA).

## Results

### Case profiles and specimens

Twenty-two strains isolated in two hospitals in Southern Taiwan (Table 2) from 1989–2010 were randomly chosen for this study. The male to female ratio was 1.4:1. Age and severe clinical manifestations were unavailable for two patients. The age range for the rest of the samples was 0.1 to 34.7 years (median, 1.8 years). Of the seven severe cases, four were younger than six months.

**Table 2:** Background data for each sequence used in this work.

VP1	3D	Isolation year	Specimen	Age (month)	Gender	Note
AB646477	AB639782	2009	RS	44	Male	
AB646478	AB639774	1989	RS	N/A	Male	
AB646479	AB639793	1989	CSF	128	Male	Aseptic Meningitis
AB646480	AB639792	2003	TS	107	Male	
AB646481	AB639791	2003	TS	45	Female	
AB646482	AB639788	2005	TS	416	Male	
AB646483	AB639787	2009	TS	30	Female	
AB646484	AB639777	1994	Blood	1	Male	Neonatal Sepsis
AB646485	AB639778	1994	CSF	48	Female	Aseptic Meningitis
AB646486	AB639779	1993	CSF	N/A	Female	Aseptic Meningitis
AB646487	AB639775	1993	TS	4	Male	
AB646488	AB639776	1993	TS	15	Male	
AB646489	AB639784	2009	RS	8	Female	
AB646490	AB639785	2008	TS	2	Female	
AB646491	AB639783	2008	TS	2	Male	
AB646492	AB639790	2009	TS	28	Male	
AB646493	AB646500	2008	RS	1	Male	
AB646494	AB639786	2010	TS	37	Female	
AB646495	AB646499	1994	CSF	2	Female	Meningoencephalitis
AB646496	AB639781	1999	CSF	1	Male	Aseptic Meningitis
AB646497	AB639780	1999	RS	6	Male	Acute hepatitis
AB646498	AB639789	2010	TS	98	Female	

CSF indicates cerebrospinal fluid; RS, rectal swab; TS, throat swab.

### Phylodynamic reconstruction of the VP1 gene

**Fig 1:**
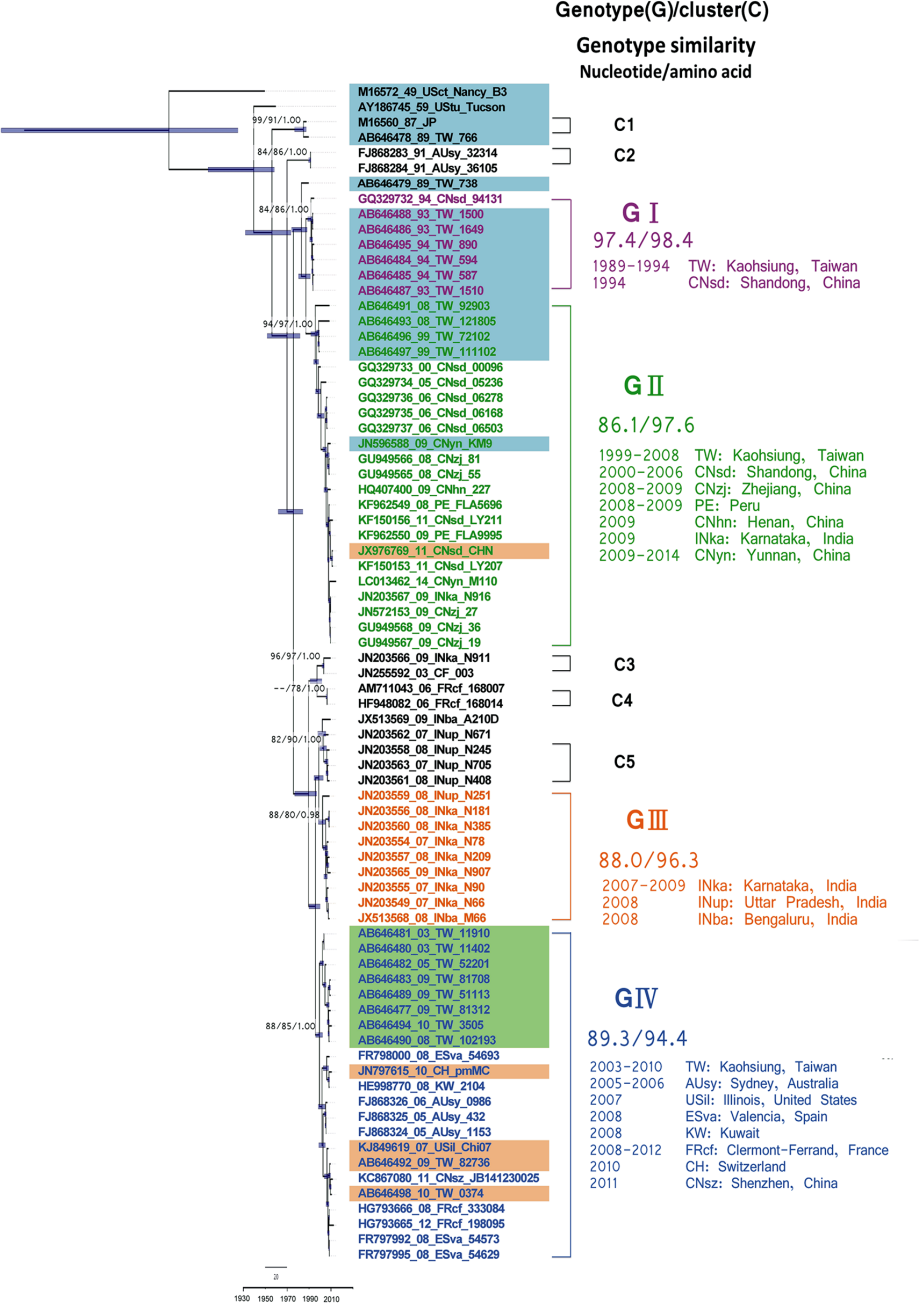
Maximum clade credibility (MCC) phylogeny of coxsackievirus B1 VP1. The full VP1 region was compared in 77 strains with prototype coxsackievirus B3 strains used as the outgroup. The tree shows the proportional relationship between branch length and time, with the time scale in years given in the bottom line. The dashed line in the time scale is the scale bar for nucleotide genetic distance. The support values for key nodes are indicated by bootstrap (BS) or posterior probability (PP) according to neighbor-joining (NJ), maximum likelihood (ML), or BEAST method and are indicated as BS-NJ/BS-ML/PP-BMCMC. Each branch thickness is also indicated as PP-BEAST. Genotypes, clusters and nucleotide/amino acid similarity within genotype are shown on the right. For each strain name, VP1 genotypes are differentiated by color (Genotype I: purple, Genotype II: green, Genotype III: orange, Genotype IV: blue), and 3D^pol^ genotypes are differentiated by shading (Genotype A: blue, Genotype B: green, Genotype C: orange).

Using an extrapolated genotype demarcation of 15% [37], the NJ, ML, and BMCMC methods VP1 trees yielded four main genotypes (designated GI–GIV) and four small clusters (C1–C4) (Fig 1). Notably, after evolving from a common ancestor, AY186745 was located alone in a separate branch. The C1 cluster was comprised of strain M16560 and one Taiwan strain (Accession no. AB646478 isolated in 1989). The C2 cluster, which was comprised of two strains isolated in Australia (1991), emerged thereafter. The C3 cluster was comprised of strains isolated in the Central African Republic (2003) and India (JN203566; 2009), the C4 was made up of strains isolated in France (2006), while the C5 was composed by strains isolated in Uttar Pradesh, India (2007–2008; Accession Nos. JN203558, JN203561, and JN203563). Further, the GI strains all appear to have been isolated in Taiwan and China (1993–1994). The GII strains were all isolated in Taiwan and China (1999–2014) except for one strain isolated in India (JN203567, 2009) and two strains in Peru (2008–2009). The remaining India strains (2007–2009) were clustered in the GIII genotype, with the exception of the five strains that did not cluster in any groups with high support values in the NJ and ML trees (Accession Nos. JX513169, JN203588, and JN203581-JN203583). The GIV genotype, which had a relatively wide geographic distribution, was comprised of strains isolated from Taiwan (2003–2010), the US (2007), Spain (2008), Kuwait (2008), the Central African Republic (2010), China (2011), and France (2008 and 2012). The phylodynamic reconstruction of these results also revealed a chronological outbreak trend in each prevalent viral cluster in Taiwan. For example, GI, GII, and GIV (major) +GII were implicated in sequential outbreaks in Taiwan from 1993–1994, in 1999, and from 2008–2010, respectively. Although all genotypes and clusters had high support values, most of the internal nodes (i.e., nodes near the roots) had low BS support values.

**Fig 2:**
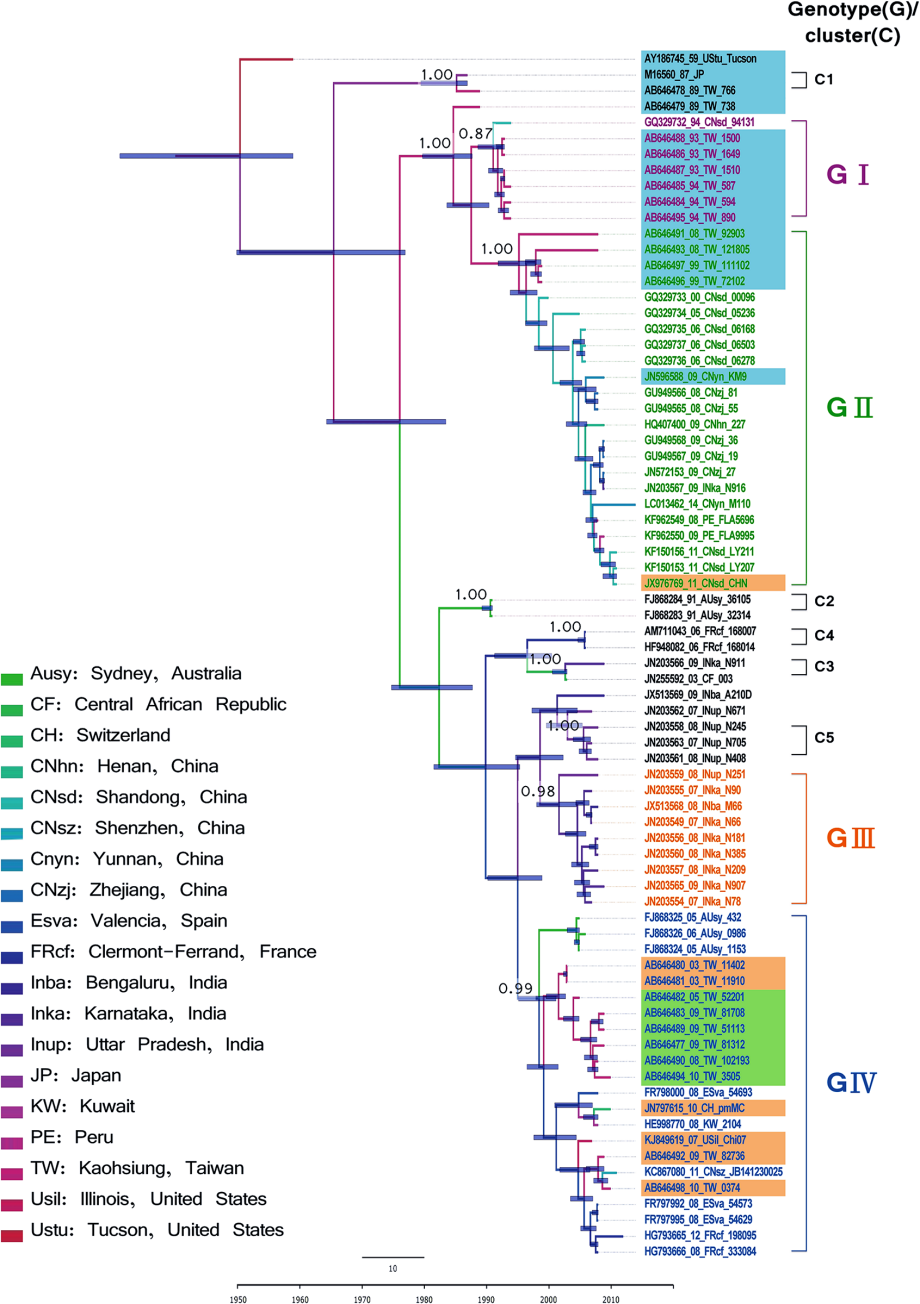
Maximum clade credibility (MCC) tree summarizing the inferred geophylogeny of 76 coxsackievirus B1 VP1 sequences. Branch thickness indicates the location probability and is colored to indicate the most probable location. The support values for key nodes are indicated by posterior probability values. The time scale in years is given in the bottom line. The dashed line above the time scale is the scale bar for genetic distance. For each strain name, VP1 genotypes are differentiated by color (Genotype I: purple, Genotype II: green, Genotype III: orange, Genotype IV: blue), and 3D^pol^ genotypes are differentiated by shading (Genotype A: blue, Genotype B: green, Genotype C: orange).

After removing the outgroup strain, the dataset was used to reconstruct spatiotemporal transmission history with the BMCMC method. This BMCMC tree shows a similar topology with and without the outgroup data (Figs 1 and 2). Notably, the topology highlighted by this tree indicates a specific spatiotemporal-structured signature, with most of the terminal branches being short. Analyses of the spatiotemporal transmission of CV-B1 using ArcGIS Explorer and the SPREAD program (Movie) revealed the following three transmission pathways with BF>3: from Taiwan to Shandong, China; from Shandong to Zhejiang; from both Shandong and Zhejiang to Yunnan.

**Fig 3:**
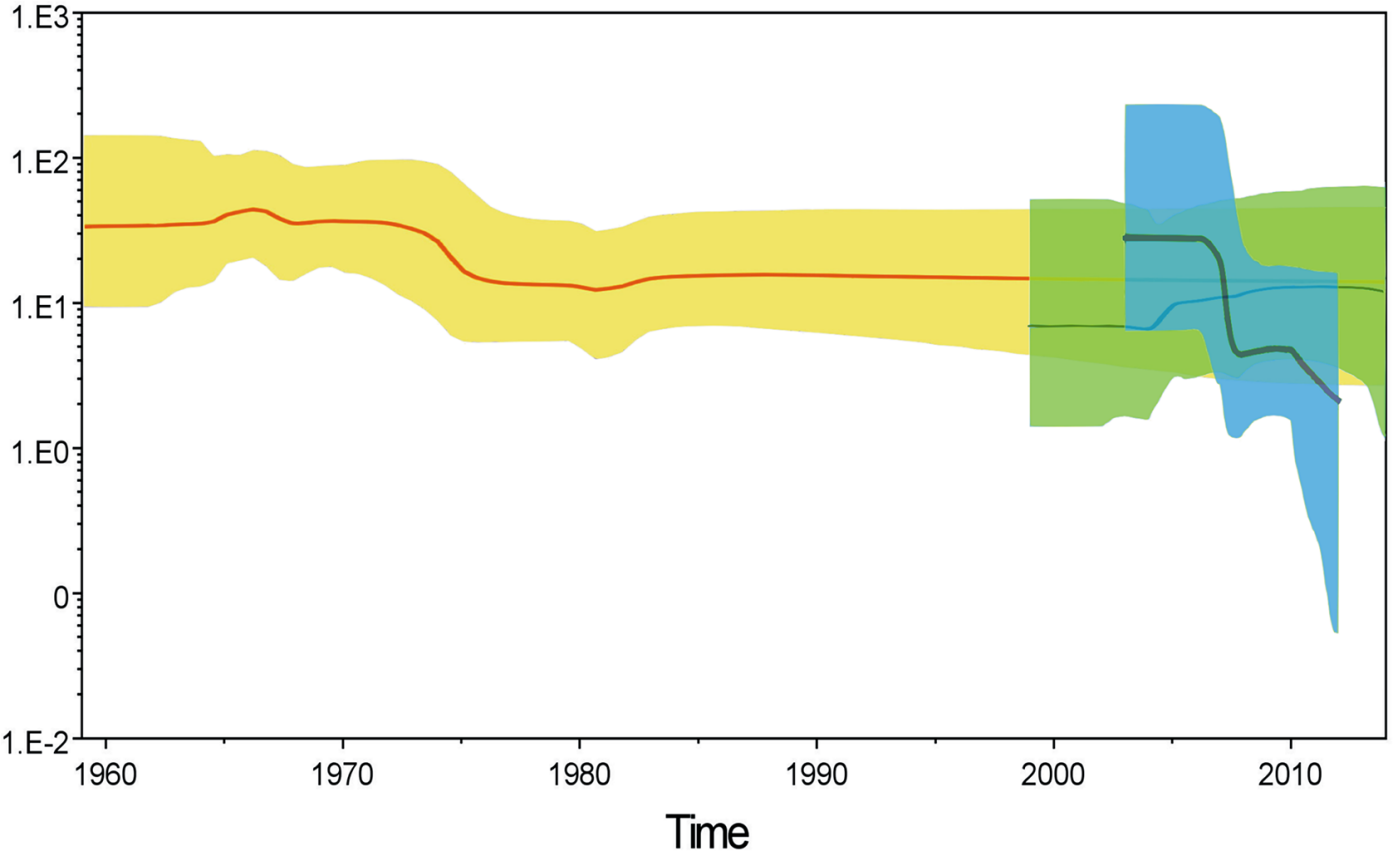
Effect of VP1 genetic diversity on virus population. A Bayesian Skyline Plot (BSP) was used to plot changes in the effective population size over time for coxsackievirus B1 and for subgenotypes II and IV. The x-axis is the time scale (years), and the y-axis is the logarithmic scale (where Ne is the effective population size and t is the generation time). The thick solid line indicates the median estimates, and the grey area shows the 95% highest probability density (HPD).

The estimated TMRCA (95% highest posterior density, HPD) was 1949 (1932–1959), with a rate of evolution of 7.73 × 10^−3^ substitutions per site per year (s/s/y). The demographic history of VP1 revealed by BSP showed that, when CV-B1 first appeared, the effective median population size was 33.0 Neτ (9.2−136.5; 95% HPD) and then dramatically decreased from 1970–1975. Since then, the population size has stabilized at 13–15 Neτ with only minor fluctuations (Fig 3). It is possible that the dip during 1970 to 1975 may have resulted from the lack of sequences reported in this period. Since strains in the GI and GIII were isolated within a short period (1993–1994 and 2007–2009, respectively), the demographic dynamics of GII and GIV the only genotypes used for further analysis. The BSP for GII indicated that this genotype first appeared in 1990 with an effective population size of 6.85. The population then increased in 2005, peaked in 2010, and then dropped to 11.8 in 2014. The GIV strains had a large population size (30.0) when they first appeared, which then showed a stair-like drop in 2005. The population size was 4.4 by 2008, and, after another drop in 2010, the population size reached 2.13 in 2012.

### Phylogenetic analysis of the 3D^pol^ gene

**Fig 4:**
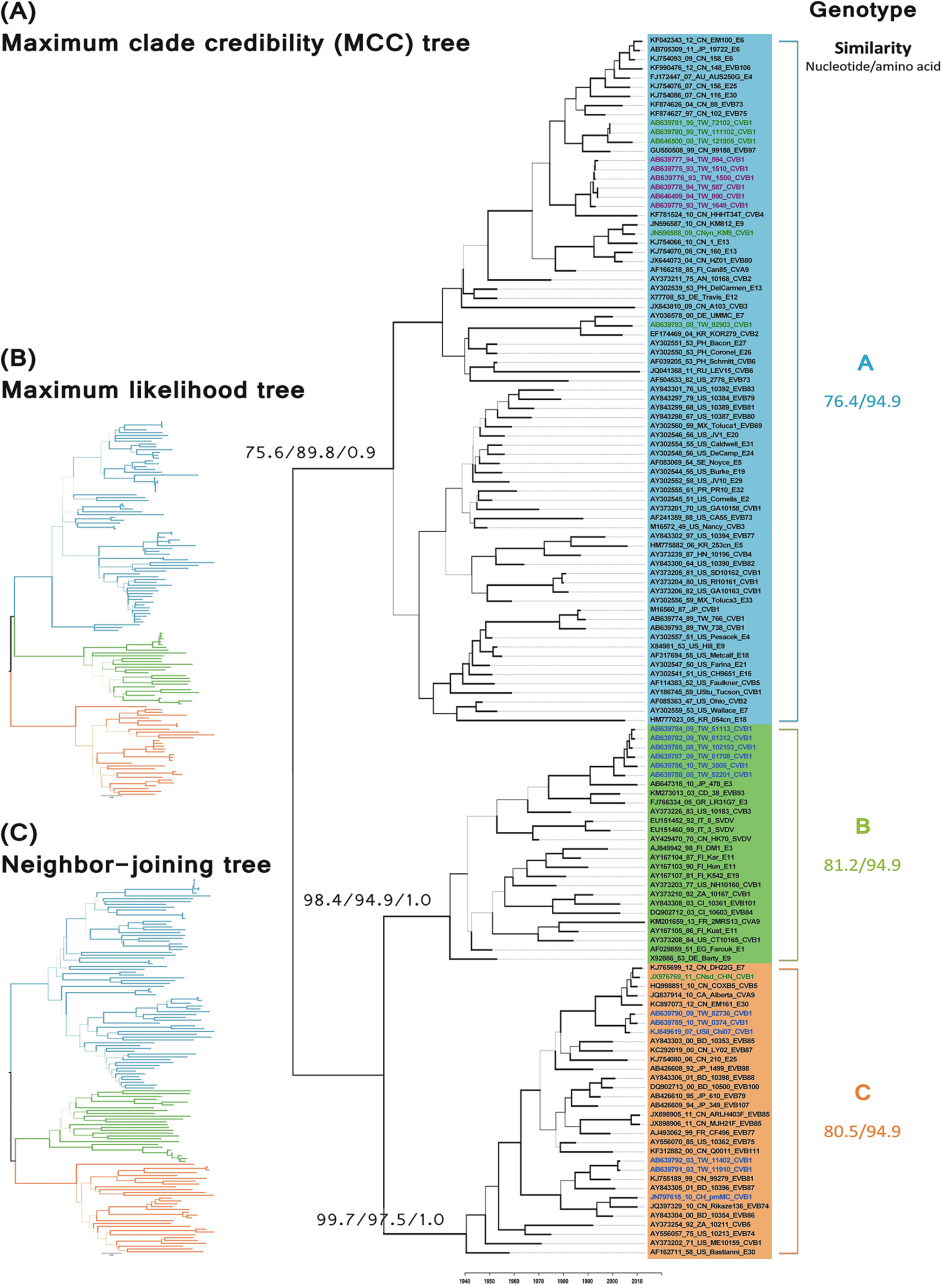
Phylogenies of enterovirus B by 3D^pol^ region. The RNA polymerase 3D^pol^ regions (nt 6682–7092) of 133 strains were compared. Branch thickness indicates the support values. The dashed line below is the scale bar for nucleotide genetic distance. (A) Maximum clade credibility tree. The support values for key nodes are indicated by bootstrap (BS) or posterior probability (PP) according to neighbor-joining (NJ), maximum likelihood (ML), or BEAST method and are indicated as BS-NJ/BS-ML/PP-BMCMC. The time scale in years is given in the bottom line. Genotypes and nucleotide/amino acid similarity within genotype are shown on the right. For each strain name, 3D^pol^ genotypes are differentiated by shading (Genotype A: blue, Genotype B: green, Genotype C: orange), and VP1 genotypes are differentiated by color (Genotype I: purple, Genotype II: green, Genotype III: orange, Genotype IV: blue). (B) Maximum likelihood tree. (C) Neighbor-joining tree. Branch is colored to indicate the genotype.

**Fig 5:**
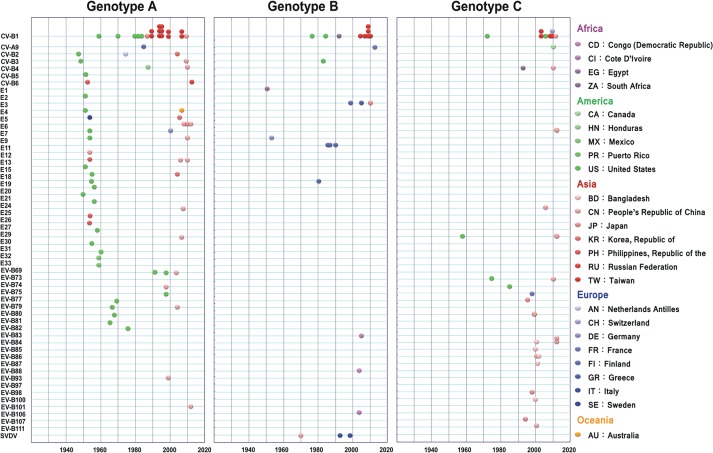
Distribution of 3D^pol^ genotypes of enterovirus B by isolation year and location. Timelines (horizontal lines) for each sampling sequence in each serotype (left vertical lines). Isolation locations for Genotypes A (left), B (middle), and C (right) are differentiated by color. The isolation countries are colored as shown on the right.

Three genotypes (GA, GB, GC) with high support values were depicted in NJ, ML and BMCMC analyses of 133 sequences of 411 nt in the 3D^pol^ region (Fig 4). Obviously, the CV-B1 strains showed a genotypic incongruence between the VP1 and 3D^pol^ regions. Briefly, the GA included several ancestral CV-B1 strains (AY168745, M16560, AB646478, and AB646479); the cluster of all VP1 GI strains; and five GII strains (AB639780, AB639781, AB643500, JN596588 and AB639783). Interestingly, strain 766 isolated in Taiwan in 1989 had a high similarity with strain M16560 in the 3D and VP1 regions (99.76% and 98.9%, respectively) and were clustered together in both trees. The GB appears to be composed of six Taiwan GIV strains (AB639782 and AB939782-8), while the GC included only one GII strain (JX976969) and five GIV strains (AB639789, AB639790, AB639791, AB939792, and KJ849619). Our phylogenetic analysis also revealed that at least two CV-B1 clusters co-circulated in Taiwan during the outbreaks that occurred from 2009–2010. All of the strains isolated from Taiwan during these outbreaks were clustered together in the GB genotype with one other strain isolated in Taiwan in 2005, with the exception of two strains that were clustered in the GC genotype with a US strain isolated in 2007 (KJ849619). Further, some CV-B1 ancestor strains were isolated in 1970–1990, but their VP1 sequences were too short for analysis in this study [25], four of them were clustered in the GA (AB373201, AB373204, AB373205, and AB373204-6), three strains in the GB (AY373210, AY373203, and AY373208), and one strain in the GC (AY373202). Globally, the spatiotemporal structures of the tree topologies showed relatively long terminal branches extending to a single strain or to a small terminal cluster, especially in the NJ tree. The similarities in nt and aa sequences among the 3D region in EV-B were 76.4–100.0% and 94.2–100%, respectively. Most ancestor strains of EV-B (isolated in 1950–70s) were clustered in GA. The exceptions were E1, E9 and SVDV in GB and E30 in GC (Figs 4 and 5).

### Detection of recombination events

**Fig 6:**
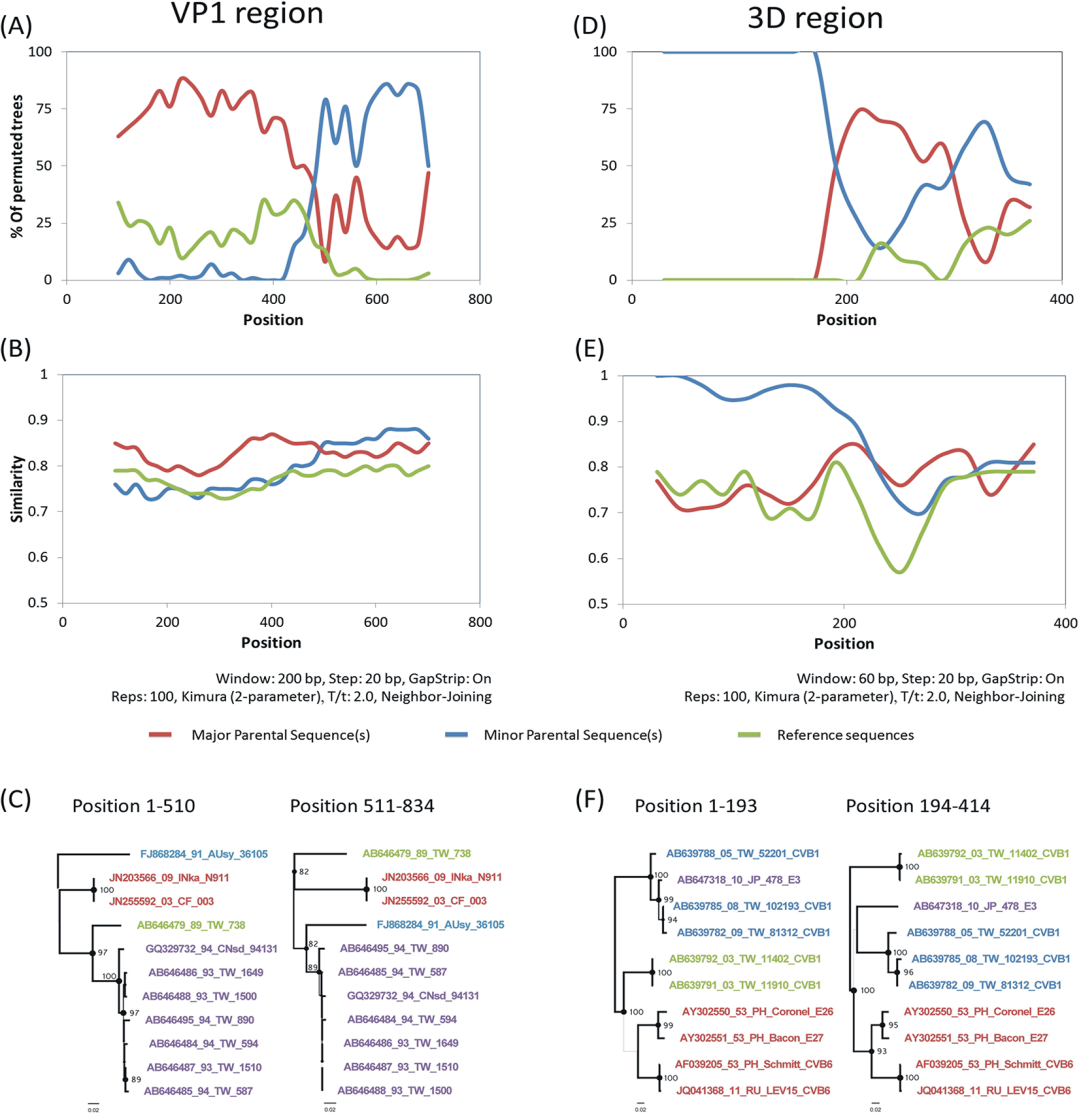
Analysis of recombination events in 76 VP1 coxsackievirus B1 and 133 3D^pol^ enterovirus B sequences. The left figures (A–C) show the results for the VP1 region while the right figures (D–F) show the results for the 3D^pol^ region. The relationships between query strains (potential recombination strains) and reference strains (major parent, minor parent, or non-donor strains) are depicted by phylogenetic distance and genetic distance in bootscan plots (A, D) and SimPlots (B, E), respectively. In both plots, which were generated with the Simplot program, potential recombination breakpoints defined where sequence crossover occurs. (C, F) Phylogenetic comparison. The trees of the fragments flanking the breakpoint showed that the recombinant strains were located in different cluster with high bootstrap value. Strain names are color coded as follows: potential recombination strains (black), major donor strains (red), minor donor strains (blue), and non-donor strain(s) (green).

Two potential recombination patterns were detected by RDP and Simplot programs, one in the VP1 and one in the 3D^pol^ regions (Fig 6). In the VP1 region, the GI strains may have resulted from a recombination event occurring between C4 (i.e., JN203566 and JN255592) and FJ868284 (1991, Australia), which was supported by Chimaera (3.43 ×10^−2^) and 3seq (2.94 ×10^−2^) in the RDP program. In the 3D^pol^ region, an interserotypic recombination (AB647318, Japan, 2010, E3) was found to be the major parents of four strains (AY302550, AY302551, AF039205, and JQ041368) and the minor parents of three Taiwan strains, all of which were CV-B1 (AB639782, AB639785, and AB639788). This pattern was supported by Maxchi (2.24 ×10^−4^) and SiSscan (6.41 ×10^−6^) in the RDP program. The recombination patterns were further supported by Bootscan and Simplot in the Simplot program. Notably, the phylogenetic incongruencies with high BS values also revealed gene fragments between breakpoints in the NJ tree.

## Discussion

In this study, we have investigated the origin, spread, and demographic history of CV-B1 from 1947 to 2012. Using our sequencing data, we were able to successfully conduct a phylodynamic analysis on both the VP1 and 3D^pol^ regions. The division of lineages into geographically or globally distributed tracts has also been reported in several other enteroviruses [38, 39]. The analysis of VP1 revealed a stair-like (unbalanced) topology, which has also been observed in several other phylogenic investigations of enterovirus strains [13, 40]. Since VP1 contains the major immune epitopes found in EV capsid, the unbalanced structure of the VP1 topology implies that a bottle-neck in its transmission occurred under continuous host immune-driven selection [41]. The sequential boom-and-bust cycles, reflected in the stair-like stem, may also be attributable to rapid divergence and turnover among the taxa as prevalent virus lineages were continuously replaced by newly emerging subclusters.

In contrast to the immune-directed evolution of the VP1 region, the grouping of the 3D^pol^ region reportedly correlates with viral isolation time rather than with serotype [22]. Here, 3D^pol^ region seems clustered together by species (at least in EV-B) and all had well-supported values. Evolution of the 3D^pol^ gene product is likely to be constrained by the essential biological function of the RdRp and the relatively stable intracellular conditions in which the product is located. In this context, a star-like topology, like that observed for 3D^pol^, is reportedly a signature of a nonstructural gene [42]. When terminal branches are long relative to internal branches in a phylogenetic topology, this has been referred to a star-like tree [41]. Notably, this star-like topology is only showed in NJ tree in this study (Fig 4). Since NJ tree is constructed by distance-based, this observation may explain the low variability and/or multiple reversions or recombinations observed within the region studied.

Only a few of the VP1 trees in this study had BS support for the internal nodes, i.e., those near the root of the phylogeny. Low support values indicate that more than one tree topology fits the dataset. A similar phenomenon has been observed previously in a partial VP1 region of CV-B1 in studies performed in the US, Korea, and China [9, 20, 43, 44] and in a partial 3D^pol^ region of enterovirus [22, 45]. Possible explanations of this include rapid evolutionary radiations among taxa, insufficient quantity of informative sites [46], or presence of chimeric genes resulting from recombination or gene flow [47]. The lack of GenBank sequences or outbreak reports does not rule out the possibility that the virus strains circulated at other times or in other locations. Since most EV infections are sub-clinical, an undetected circulation of an EV-lineage in a distinct region is highly possible. For other enteroviruses often involved in outbreaks, GenBank samples are readily available. For example, as of 2015, GenBank has 4000 full EV-A71 VP1 sequences, about 2000 poliovirus 1 sequences, and about 1000 echovirus 30 VP1 sequences. In contrast, the number of CV-B1 samples available in GenBank is relatively small (<100) since CV-B1 outbreaks and severe infections were rare until 2007. Samples for the 3D^pol^ region are even rarer. Further, older sequence data are also limited. Although the US Centers for Disease Control and Prevention has a long tradition of infectious pathogen surveillance, almost all older sequences (pre-1980) from the US were too short to include in this study. Thus, the low BS values in the internal nodes and long terminal branches observed in this study suggest that some clades were not detected in earlier time periods. There has been some debate about the use of PP values, which are commonly higher than corresponding BS frequencies, but recent data suggests that PP-BMCMC is, in most cases, a less biased predictor of phylogenetic accuracy compared to BS values [48]. The most problematic aspect of using BS values to gauge the accuracy of the phylogeny is that evolutionary complexity cannot be estimated with a simple model. The Bayesian analysis combining substitution, clock model, and population model, markedly increased the number of well-supported nodes.

The phylogenetic relationships in CV-B1 have been reported in previous studies performed in the US, Korea, and China [9, 20, 43, 44]. Despite their differences in grouping assignments, previous reports have tended to focus on four main clusters (A-D). Briefly, ancestor strains isolated in the US in the 1980s can be grouped into two clusters. For example, Kim et al. and Baek et al grouped strains into clusters A and B [9, 44]. However, Zhang et al. grouped strains into clusters A and C [20]. Cluster C in Kim et al. and in Baek et al. corresponded to lineage D in Zhang et al. and GII in current study. Further, cluster D in Kim et al. and in Baek et al. correspond to lineage B in Zhang et al. and GIV in the current study. Strains JX976769 and JN797615 have been identified as recombinant strains based on full-length genomes [20]. However, their break points were neither detected in the VP1 region (positions: 2452–3260 of M16560) nor in the 3D region (6682–7092). Therefore, JX976769 is the only GII strain clustered in GC in the 3D tree in this study, which is due to a recombination event.

In addition to these discrepancies in genotype nomenclature in the literature, the identity of the sequence with GenBank accession number M16560 is also controversial. Many previous works have designated this sequence a prototype strain of Conn-5, which was isolated in 1948 [9, 43, 44, 49]. However, a recent study designated M16560 as a strain isolated in Japan in 1980s [20]. Since M16560 has high nt similarity with Taiwan strain 766 (1989), in both the VP1 and 3D^pol^ regions, in this study, the virus strain was designated as a Japanese strain, and the isolation year was used as the publication year. Additionally, a relaxed clock model was used in this study to allow rate variation among lineages or branches.

## Conclusion

Thus, while these various issues (i.e., the abundance of sequence data, genotype nomenclature, strain naming, etc.) posed major limitations, we believe that the data described here present the most thorough, all-encompassing phylodynamic analysis of CV-B1 outbreak behavior. In this study, we have reconstructed the spatiotemporal transmission and population dynamics for this virus, allowing the detections of specific recombination events in the VP1 and 3D^pol^ regions. The BMCMC tree for VP1 also showed that CV-B1 evolved from a common ancestor and then co-evolved and co-circulated chronologically. Some outbreaks involved geographically clustered genotypes, such as GI and GIII. The fittest clusters, GII and GIV, remained prevalent for 2–4 years, resulting in a ladder-like backbone topology. Although each cluster reveals a different spatiotemporal trend, different subclusters may have co-circulated in the same geographic location. In contrast to the relatively stable demographic history of CV-B1, GIV revealed a sharp decrease in population size in 2009. The GII population decreased during the same period, but its decrease was more gradual. Generally, the GI and GII strains in the VP1 region corresponded to GA genotype in the 3D^pol^ analysis, whereas the GIV strain in VP1 was clustered in GB and GC. Understanding the population dynamics and epidemic outbreaks of a virus in terms of pathogen evolution, host immunity, and transmission, provides additional insight into the biology of CV-B1 itself and provide data (e.g., the BSP results) that are useful for forecasting potential outbreak trends.

## Supporting Information

S1 Movie Spatiotemporal transmission of coxsackievirus B1. Pushpins show the locations of sampling sites, and the size of the circle indicates the number of lineages in the location during the given time duration. The lines between locations show the transmission route, and the opacity level indicates the support value of the node. The movie was created using ArcGIS Explorer Desktop (ESRI).
(MP4)
